# Supplementary material for: Chlorine Modulates Photosynthetic Efficiency, Chlorophyll Fluorescence in Tomato Leaves, and Carbohydrate Allocation in Developing Fruits
Source: Int J Mol Sci. 2025 Mar 24;26(7):2922. doi: 10.3390/ijms26072922 (PMC11988456; doi:10.3390/ijms26072922)
Supplement: Supplementary file 1 [file ijms-26-02922-s001.zip › ijms-3466381-supplementary.pdf]

Supplement Tables and Figures

Table S1 Effects of different concentrations of chloridion on stem diameter. CK (0 mmol·L^{-1} Cl^-), T1 (1 mmol·L^{-1} Cl^-; $CaCl_2$), T2 (2 mmol·L^{-1} Cl^-; $CaCl_2$) and T3 (3 mmol·L^{-1} Cl^-; $CaCl_2$). Data are presented as mean ± SD (n = 4). Different letters indicate significant differences at $P < 0.05$.

Treatments	CK	T1	T2	T3
21d	13.10±0.65a	13.25±0.42a	12.60±0.25a	12.87±0.68a
35d	12.11±1.26a	12.93±0.60a	12.24±0.53a	12.55±0.98a
49d	12.35±0.72a	12.68±0.30a	12.61±0.65a	12.47±0.46a

Table S2 Effects of different concentrations of chloridion on SPAD of tomato plants.

Treatment / Day	CK	T1	T2	T3
21d	39.86±0.16c	41.34±0.91bc	42.94±1.60ab	44.19±0.98a
35d	39.93±2.11c	42.36±1.61abc	42.26±1.21abc	43.10±0.19a
49d	40.00±1.67c	46.38±0.17a	43.13±1.73ab	45.51±0.55a

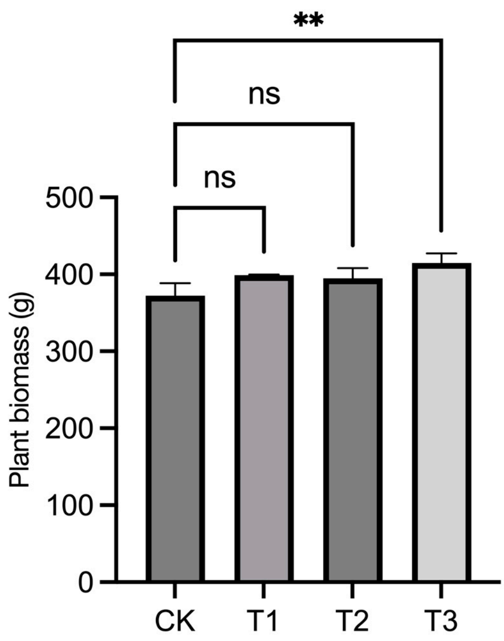

Figure S1 Effects of different concentrations of chloridion on biomass of tomato plants. Data are presented as mean ± SD (*n* = 3). Levels of significance: P > 0.05 (ns, not significant), and ∗∗P ⩽ 0.01.

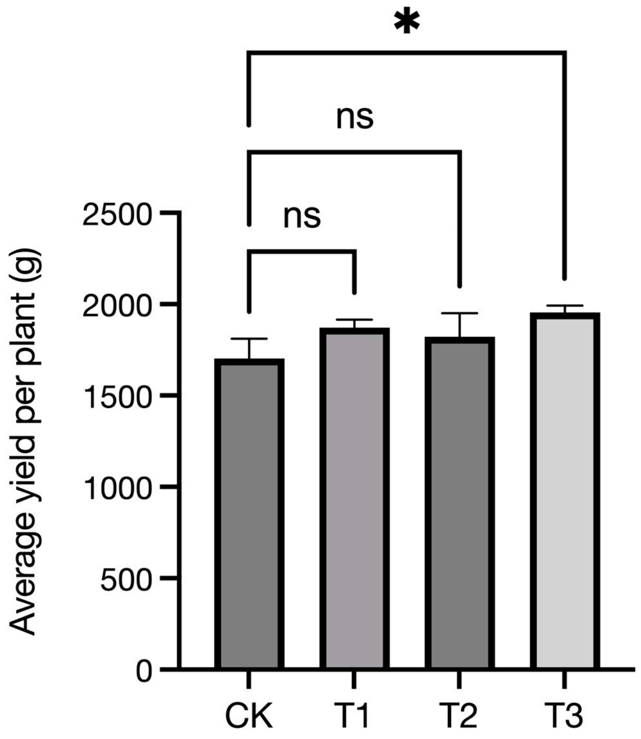

Figure S2 Effects of different concentrations of chloridion on yield of plants. Data are presented as mean ± SD (*n* = 3). Levels of significance: P > 0.05 (ns, not significant), and ∗P ⩽ 0.05.
